# Use of electrochemically activated aqueous solutions in the manufacture of fur materials

**DOI:** 10.1186/s40064-016-1784-6

**Published:** 2016-02-29

**Authors:** Anatoliy G. Danylkovych, Viktor I. Lishchuk, Oksana O. Romaniuk

**Affiliations:** Department of Technology, Leather and Fur, Kyiv National University of Technologies and Design, Str. Nemirovich-Danchenko, 2, Kiev, 01011 Ukraine

**Keywords:** Electroactivated aqueous solution, Anolyte, Catholyte, Raw furs, Soaking, Degreasing, Tanning

## Abstract

The influence of characteristics of electrochemically activated aqueous processing mediums in the treatment of fur skins with different contents of fatty substances was investigated. The use of electroactive water, namely anolytes and catholytes, forgoing antiseptics or surface-active materials, helped to restore the hydration of fur skins and to remove from them soluble proteins, carbohydrates and fatty substances. The activating effect of anolyte and catholyte in solutions of water on the processes of treating raw furs is explained by their special physical and chemical properties, namely the presence of free radicals, ions and molecules of water which easily penetrate cells’ membranes and into the structure of non-collagen components and microfiber structure of dermic collagen. The stage of lengthy acid and salt treatment is excluded from the technical treatment as a result of using electroactivated water with high oxidizing power. A low-cost technology of processing different kinds of fur with the use of electroactivated water provides for substantial economy of water and chemical reagents, a two to threefold acceleration of the soaking and tanning processes and creation of highly elastic fur materials with a specified set of physical and chemical properties. At the same time the technology of preparatory processes of fur treatment excludes the use of such toxic antiseptics as formalin and sodium silicofluoride, which gives grounds to regard it as ecologically safe.

## Background

The modern manufacturing processes must comply with stringent requirements with regard to the efficiency of the use of raw materials and chemical reagents, the decrease in energy consumption and the enhancement of technical processes and sustainability. This is especially relevant for chemical production which presupposes the use of large amounts of processing mediums with a strong concentration of chemical reagents, including toxic ones. This directly concerns the manufacture of fur and leather products.

In view of this, efforts are taken in order to reduce costs and replace environmentally hazardous chemicals with new reagents, especially at the initial stage of processing raw materials. New operating procedures with positive physical and chemical impacts, including the influence of the ultrasonic field, are developed Thus, at the stage of the soaking of leather, researchers sed alkaline proteases and optimized the latter’s activity with regard to pH and temperature. A substantial decrease in oxygen consumption for effluent treatment has been recorded. The researchers (Saran et al. 2013) studied the effect of 2–5 % protease on the dehairing and the effect of 5–10 % lipase on the degreasing of animal skins. The authors concluded that the application of these ferments is environmentally safe, but their use over a prolonged time (8–12 h) should be restricted in view of the possibility of partially destroying skin collagen and decreasing the mechanical and physical properties of skins.

The effectiveness of the ultrasonic field in the soaking and degreasing of skins is highlighted in the research (Morera et al. 2013; Sivakumar et al. 2009). An increase of up to 23 % in water absorbability of steerhide was revealed, coupled with a 36 % decrease in the duration of the soaking of skins, which had been aid-dried. By using ultrasound the researchers (Sivakumar et al. 2009) achieved a twofold cut in the duration of degreasing under production-line conditions in comparison with control treatment. The technology advanced by them requires complex manufacturing equipment (Ma et al. 2014).

The existent technologies of treating furs depending on their structure, require a 3- to 20-fold higher consumption of water (Danilkovich 2011; Danylkovich et al. 2009) in comparison with leather production. In particular, the consumption of water for stages of soaking and air-drying per one ton of raw material is: rabbit skins—9 m^3^, sheepskin and fox—17 and 25 m^3^ respectively, at the stage of dying the skins with oxidizing semiproducts—175 m^3^, which includes multiple rinsing.

One of the ways to enhance sustainability of fur production and to reduce consumption of water for processing mediums could be its activation by different methods, in particular, by the magnetic field, ultrasound, jarring action, heat treatment (heating, cooling, freezing and defrosting) and electrochemical activation (ECA) as a result of which water acquires anomalous properties (Fidaleo and Moresi 2006; Plutakhin et al. 2013). The physical and chemical properties of ECA water and solutions of chemical reagents are largely accounted for by their declustered structure, high oxidation-reduction potential (ORP) and thermodynamic disequilibrium (Hsu and Kao 2004; Kim et al. 2000; Shirahata et al. 2012), which is sustained over an extended period of time. It was ascertained that ECA water accelerates biotechnological processes (Aider et al. 2012).

The study of the effect of ECA water on the structure and properties of biopolymers allowed to establish that the use of one of its phases, namely anolyte, which has a germicide effect on the florula of protein-based raw materials (Cloete et al. 2009; Robinson et al. 2010), allows to exclude antiseptic reagents from the technical process. The germicidal properties of ECA water were tested on plant and animal models, in particular, hydra and ceriodaphnia dubia (Goncharuk et al. 2005).

The study (Pankiv et al. 2013) highlights the characteristics of anolyte and catholyte and their influence on the management of fermentation and genetic activity of distillers’ yeast. At the same time it is recommended to use ECA water for standardizing alcoholic beverages produced by different companies. A positive effect of activated water was observed in the synthesis of furfural (Kashkovskiy and Kamenskikh 2012) from vegetable waste with high content of pentosans. The use of ECA water with low pH as an active catalyst makes it possible to exclude acids from the technical process.

Since the preparatory stages of processing leathers and furs involve the use of chloride of sodium in various concentrations as well as sodium carbonate, bicarbonate and sulfate, the effect of EC water on the physical and chemical properties of these reagents is of great interest. The study (Lee et al. 2006) shows that ECA water with a negative ORP easily penetrates cells, whereas catholyte with high value of pH promotes effective release of lipids, glucosamineglycans and spheroproteins from protein-based raw materials.

The use of ECA water in drying and watering processes in the production of leather used for the uppers of footwear allows for increasing the surface area of the output material due to the plasticization effect on its structure as opposed to industrial water. This is accounted for by an increase in the deformation capacity of the material and the relaxation of tension during the drying. In particular, using catholyte enhances the deformation of samples by 1.5–5.0 % (Lutsyk et al. 2005; Zlotenko et al. 2008) depending on the type of leather. At the same time, the pH and ORP stability of electroactivated water after boiling (Bovsunovskyi et al. 2005) allows for using it in making the upper in shoe manufacture. In order to increase the plasticity of fur skins and leather in the process of their production it is recommended that the semifinished product is treated with solutions of catholyte (Zorina and Zeleva 2002; Zorina et al. 2002) with low concentrations of benzyl alcohol, glycerin and oxalaldehyde.

An analysis of literary sources testifies to the relevance of research on the use of ECA aqueous solutions in mass exchange processes of manufacturing leather and furs. The use of activated water in the treatment of protein-based raw materials can be effective provided high stability of the characteristics of electroactivated aqueous solutions and chemical activity in the reactions of interaction with non-collagen components of animal skins.

In the upgrading of existent technologies of processing furs and the elaboration of new ones, especially at the stages of preparation, which foresee a lengthy production run and substantial amounts of aqueous solutions with chemical reagents, the problem of enhancing processing treatment is especially relevant. One of the ways of solving this problem is the use of electrochemically activated industrial water and establishment of the efficiency of the preparatory stages of fur production.

## Methods

The study is aimed at investigating the influence of electroactivated water on the efficiency of the main processes of fur production. Two fractions of electrochemically activated water will be used, namely anolyte and catholyte. The technology foresees implementation of a set of consecutive physical and chemical processes and mechanical operations of raw materials treatment. They include watering of the skin tissue for restoring the water content lost in skin curing, the removal of hypoderm, degreasing, acid and salt treatment, chemical structuring, i.e. the tanning with chromium compounds, plasticization of skin tissue, i.e. greasing and drying and wetting treatments.

In order to prepare processing mediums of chemical reagents we used distilled water with the addition of 0.2 g/l of sodium chloride for electric activation. Anolyte and catholyte were obtained with a pH range of 2.8–4.0 and 9.5–11.5 in the electric water activator of the research and production company Ekovod, TOR U29.1-1285006876.001-2000 by using a silicon anode and steel cathode and ion-exchanger membrane made of cotton of special weaving (Apukhovskiy et al. 2002; Potapenko et al. 2006).

Enhanced chemical activity of declustered aqueous systems and processing mediums on their basis was explained by a number of electrochemical processes under the influence of electric current on water in the presence of an electrolyte. The water is ionized with the creation of a substantial number of highly active ionic radical elements, in particular (Prilutskiy and Bakhir 1997):

$${\text{OH}}^{ - } ,{\text{H}}^{ + } ,{\text{H}}^{ \cdot } ,{\text{O}}^{ \cdot } ,{\text{OH}}^{ \cdot } ,{\text{HO}}_{2}^{ \cdot } ,{\text{HO}}_{2}^{ - } ,{\text{H}}_{3} {\text{O}}^{ \cdot } ,{\text{H}}_{3} {\text{O}}^{ + } ,{\text{H}}_{3} {\text{O}}^{ - } ,{\text{Cl}}^{ \cdot } ,{\text{ClO}}^{ - } ,{\text{ClO}}_{3}^{ - } ,{\text{HCO}}_{2}^{ - } ,{\text{C}}_{2} {\text{O}}_{4}^{2 - }.$$

The principal reactions that occur in the electric water activator are as follows. On the anode:

$$2{\text{H}}_{2} {\text{O}} - 4e^{ - } \to {\text{O}}_{2} + 4{\text{H}}^{ + } ;\quad 2{\text{Cl}} - 2e^{ - } \to {\text{Cl}}_{2} .$$

On the cathode:

$$2{\text{H}}_{2} {\text{O}} + 2e^{ - } \to {\text{H}}_{2 } + 2{\text{OH}}^{ - } ;\quad 2{\text{H}}_{2} {\text{O}} + 2{\text{Na}}^{ + } + 2e^{ - } \to 2{\text{NaOH}} + {\text{H}}_{2} .$$

**Fig. 1:**
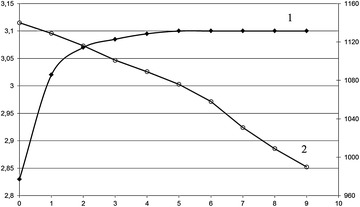
Kinetics of change of pH of anolyte (*1*) and its ORP (*2*)

The examination of physical and chemical properties of the obtained anolyte (Fig. 1) shows a slight increase in its pH and a decrease in its ORP during its storage in a hermetically sealed container for 9 days. Similar effects were observed earlier (Kashkovskiy and Kamenskikh 2012). It is necessary to note that the main changes in the pH of activated water occur in the first 2 days of storage, whereas its ORP decreases continuously. This can be explained by the neutralization of H^+^ ions in their interaction with oxide compounds formed in electrochemical reactions. The obtained results testify to the anolyte’s high stability during storage. Water can be used in the technical process immediately after its activation by flow-through cylindrical electric activators (Plutakhin et al. 2013). Unprocessed nutria and rabbit skins were chosen for treatment by air-drying. Nutria skins had fat content 22.3 % and surface area 20–24 dm^2^ (GOST standard 2916-84), whereas rabbit skins, which have higher density than those of nutrias, were 0.7–1.0 mm thick (GOST 2136-87).

The pH values of catholyte and anolyte were measured on the device pH-340. ORP was calculated by a potentiometric method using platinum and silver-chloride electrodes. The watering of the skin tissue of raw fur was assessed by gravimetric method using AXIS (Lund, Sweden) counterbalance of AD200 model.

Nutria skins were subjected to soaking with the use of a non-ionic SAMs (TOR 2484-014-22284995-99, code specification (CS-) 23, statistical acceptance test (SAT-) 50, which were dissolved in activated and distilled water of 19–21 °C and 900 % of water-to-skins mass ratio. The SAMs for soaking and degreasing were concentrated at 0.5 and 2.0 g/l respectively.

The tanning of rabbit and nutria skins of the control variant was carried out with the use of spent solution of anolyte including potassium alum KAl(*SO*_4_)_2_·12*H*_2_*O* (GOST standard 4329-77) and basic chromium sulphate ${\text{Cr}}_{2} ({\text{SO}}_{4} )_{n} ({\text{OH}})_{6 - 2n}$ with base strength 39 % and water-to-skins mass ratio of 900 %, tanning agent, g/l: potassium alum 10, chromium sulphate expressed in terms of *Cr*_2_*O*_3_**—**1.6 for rabbit skins and 1.0 for nutria skins. The base strength of the tanning agent was increased to pH 3.7–3.9 by adding a solution of sodium carbonate twice with an interval of 15 min.

The obtained results were evaluated with the use of the testing site of JSC *Chynbar* according to the methods of processing raw furs (Romaniuk et al. 2013; Savchenko et al. 2011; Statsenko et al. 2012), which presupposed the use of anolyte and catholyte. Nutria skins were soaked in anolyte and degreased in catholyte at 28–32 °C without the use of additional reagents. After degreasing, nutria skins were rinsed, pressed and treated with anolyte twice in order to prepare them for the next tanning. Rabbit skins were subjected to chrome tanning at 18–20 °C after soaking in the spent solution of anolyte and removal of hypoderm.

For comparative analysis we used fur skins obtained under existent technologies (Danylkovich et al. 2015), which presupposed the treatment of raw fur at the following temperatures, °C: soaking of nutria and rabbit skins—30 and 38 respectively; degreasing (for nutria skins)—38, pickling and tanning—30 and 40. The following expenditure of chemical agents was foreseen for treatment of rabbit and nutria skins respectively, g/l: chloride of sodium—140 and 130; SAMs—3.5 and 5.0; 40 % formalin—1.0 and 0.5, sodium fluorosilicate—0.8 and 1.5, sodium thiosulphate—17.0 and 8.0, tanning agents—16.4 and 14.0. The drying and wetting technical processes and the operations involving the semi-finished product were carried out according to the existent requirements of treating rabbit and nutria skins.

The physical and chemical features were identified according to methodology (Danylkovich and Chursin 2015). The strength of skin tissue at breaking point was measured on a strength-testing machine PT-250 M with the speed of sample deformation of 80 mm/m. Hydrothermal stability (T_HS_) of the fur skins was measured at temperature of the start of sample shrinkage during its heating in water at the rate of 2–3 °C/m.

## Results and discussion

The process of watering of fur skins in an electroactivated solution of anolyte may include a reaction between hydrogen protons and negatively charged carboxyl groups of collagen, and in case of catholyte—carboxyl groups with amines of collagen.

Under existent technologies, the sources of *H*^+^ i *OH*^−^ ions are dilute acids and alkali. When electroactivated water of anolyte is used, ionic links between the amines and carboxyl groups of R-radicals of polypeptide chains are broken as a result of which amines of the biopolymer (when anolyte is used) retain their positive charge, whereas ionized carboxyl groups are discharged. This leads to an increase in the degree of hydration of skin tissue collagen due to an ion–dipole bond and coulomb repulsion, which leads to an increase in the degree of watering of fur skins. With the use of catholyte as opposed to anolyte the watering of fur skins consists in the fact that the soaking process is accelerated due to neutralization of ionized amines of collagen.

**Fig. 2:**
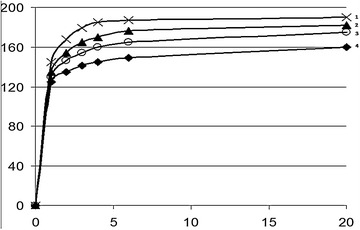
Kinetics of watering nutria skin tissues with the use of: catholyte (*1*, *2*), distilled water (*3*, *4*), SAS CS-23 (*2*, *4*) and SAS SAT-50 (*1*, *3*)

The examination of the influence of electroactivated aqueous solutions of catholyte with only 25 % SAM content used for increasing the degree of watering the skin tissue as compared to existent technologies of treating nutria skins (Fig. 2) suggests that the water lost by it in curing is restored at a considerable speed. Most of the aqueous solution is absorbed in the first hour or two. Then this process slows down and the mass of skins is increased only by 10 % in the next 20 h. This may testify to a redistribution of the diffused aqueous solution in the fibers of the skin tissue, during which the processing medium passes from the large pores into the inter-fiber and microfiber spaces of the fine structure of the collagen skin tissue. The use of catholyte, as compared to distilled water (the control variant) accelerates the processing medium’s in-diffusion into the structure of skin tissue. The SAM SAT-50 solution in catholyte was the most effective, 18 % more so than the control variant, which presupposes the use of SAM CS-23 with distilled water of 2 g/l concentration (Curve 4).

The parameters of the solutions used for soaking and degreasing are listed in Table 1, which shows that pH of spent anolyte increases and pH of spent catholyte decreases by 1.3 and 1.5 times respectively, whereas pH of the SAM solution in distilled water changes insignificantly. This testifies to enhanced chemical activity of aqueous solutions of distilled water and profound structural changes of the skin tissue and raw fur. The substantial differences in the conductivity of SAM solutions in catholyte and anolyte from distilled water testify to enhanced chemical activity of electroactivated solutions, especially of catholyte. This is also confirmed by higher conductivity of the original solutions, especially of anolyte and catholyte, 3.9 and 1.8 times respectively, as compared to distilled water. The conductivity of the spent catholyte solution is increased 2.1-fold, and 2.4-fold as compared to distilled water.

**Table 1:** Physical/chemical properties of eletroactivated aqueous solutions after degreasing of fur with SAM SAT-50

Parameter	Activated solution		
	Anolyte	Catholyte	Distilled water
pH	2.8 ± 0.1/3.7 ± 0.1	10.7 ± 0.2/6.9 ± 0.1	6.7 ± 0.1/6.1 ± 0.1
Electric conductivity (μS/cm)	2800 ± 90/2640 ± 80	1270 ± 40/2760 ± 90	710 ± 20/1160 ± 40

The numerator and the denominator are the initial and final values of water solutions

The results of the process of degreasing nutria skin tissue with a SAM solution in catholyte are listed in Table 2. The analysis of nutria skin tissues after treatment allowed to appreciate a higher effect of degreasing of skins with the use of catholyte and SAM SAT-50 as compared with the use of distilled water. Moreover, 60 % of fatty substances were removed in the first soaking, which surpassed the control variant by 13.7 %. More effective degreasing of nutria skin tissues is achieved by a higher degree of its watering with the use of SAM solution in catholyte.

**Table 2:** Content of fatty substances in skin tissue after its soaking and degreasing

Treatment	Water	SAM	Content of greasy substances, %, after the process of		
			Soaking 1	Degreasing	Soaking 2
1	Catholyte	CS-23	9.71 ± 0.21	3.32 ± 0.05	3.07 ± 0.05
2	Catholyte	SAT-50	8.97 ± 0.19	3.25 ± 0.05	3.12 ± 0.05
3	Distilled	CS-23	13.20 ± 0.27	4.09 ± 0.07	3.71 ± 0.06
4	Distilled	SAT-50	10.11 ± 0.23	3.66 ± 0.06	3.25 ± 0.05

An increase in the chemical activity of electroactivated processing mediums is observed in the structuring of collagen of nutria skin tissue during their tanning with chromium compounds (Danylkovich et al. 2015). As the results (Table 3) obtained in the consecutive processes of nutria skins treatment suggest, their structuring is increased with the use of SAM solution in catholyte. The T_HS_ of skin tissue is enhanced as a result of more intensive removal from it of non-collagen formations, including fatty substances, under the influence of active products of the electrochemical reaction that are present in the catholyte. This contributes to increased diffusion of tanning chromium compounds into the skin tissue and their subsequent efficient interaction with ionized carboxyl groups of polypeptide chains of collagen macromols. This effect is manifested to a greater extent in case of using non-ionic SAM SAT-50 as part of a degreasing solution. It is necessary to note a certain decrease of T_HS_ after acid-salt treatment as a result of the enhancement of the electrostatic effect of repulsion between the amines of macromols of skin tissue collagen.

**Table 3:** Hydrothermal resistance, °C, of nutria skin tissue after consecutive treatments

Treatment	Physical and chemical process		
	Degreasing, soaking	Acid-salt treatment	Structuring
1	58 ± 1	56 ± 1	76 ± 1
2	58 ± 1	56 ± 1	79 ± 1
3	57 ± 1	54 ± 1	72 ± 1
4	57 ± 1	54 ± 1	73 ± 1

Thus it was established that the use of processing mediums based on electroactivated water and lesser quantities of chemical reagents compared to the existent technology of processing fur provides for effective formation of the structure of skin tissue with hydrothermal resistance in accordance with the active standard. Such treatment of fur, even without the use of antiseptics, helps to preserve the bond of pillage with the skin tissue even after degreasing.

### Approbation of results of the research

The results of the research of the influence of electroactivated aqueous solutions of anolyte and catholyte on the processes of nutria fur treatment were used in the fur production at the experimental shop of JSC Chynbar enterprise, Kyiv. In view of the high chemical activity and specific physical/chemical properties of anolyte and catholyte, they were used in the treatment of furs without SAMs at different stages of the technology cycle.

At the stages of soaking, acid-salt treatment and tanning and greasing, we used anolyte solutions with pH of 2.8–4 and for degreasing—catholyte solutions with pH of 9.5–11.5. After drying and wetting processes, the fur skins were refined.

The nutria skin samples obtained in the study (Table 4) testify to the fact that skin tissue with optimal physical/chemical properties is formed with the use of anolyte with pH of 2.8–3.0 and catholyte of 9.5–10.0. An increase in pH of anolyte (option 2) leads to decreasing the strain capacity of samples, which positively affects subsequent treatments, namely tanning and greasing. Compared with the samples of the control variant, except for option 3, nutria skins treated with activated aqueous solution without SAMs have higher strain capacities of hydrothermal resistance, breaking stress of the skin tissue and somewhat higher indicators of content of unconnected greasy substances after the process of greasing.

**Table 4:** Physical/chemical properties of nutria skins

Parameter	Treatment no.			
	1	2	3	Test
pH of anolyte	2.9 ± 0.1	3.4 ± 0.1	3.9 ± 0.1	–
pH of catholyte	9.7 ± 0.2	11.2 ± 0.3	10.7 ± 0.3	–
Hydrothermal resistance after tanning (°C)	65.0 ± 1	69.0 ± 1	59.0 ± 1	63.0 ± 1
Mass content, (%) in the skin tissue of unconnected fatty substances after degreasing	3.62 ± 0.06	3.8 ± 0.06	3.3 ± 0.05	4.1 ± 0.07
After greasing	17.4 ± 0.4	18.3 ± 0.5	17.9 ± 0.4	16.2 ± 0.4
Chrome tanning agent on Cr_2_O_3_ basis	0.89 ± 0.02	0.83 ± 0.02	0.90 ± 0.02	0.93 ± 0.02
Breaking stress at the tear of an entire skin (N)	65.0 ± 3.3	64.0 ± 3.3	58.0 ± 3.0	62.0 ± 3.1
Total strain (%)	15.0 ± 1.4	13.2 ± 1.2	10.4 ± 0.9	12.7 ± 1.1
Frozen strain (%)	8.3 ± 0.6	9.7 ± 0.7	6.0 ± 0.4	6.2 ± 0.4

The volumes of fatty substances and chrome oxide are based on a totally dry substance

A decrease in hydrothermal stability and breaking stress given an over 3.5-fold increase in the pH of anolyte is caused by uneven distribution of chromium (iii) compounds in the skin tissue as a result of their (mostly) superficial interaction with carboxyl groups of polypeptide chains of collagen.

In order to reveal the influence of fur skins’ density on the process of treatment as well as its properties we obtained samples of wool breed rabbit of average thickness. They were not subject to degreasing due to substantially lower content of fatty substances in the skin tissue as compared to nutria skins. The results of these skins’ test are listed in Table 5.

**Table 5:** Physical/chemical properties of wool breed rabbits as treated by anolyte

Parameter	Treatment no.			
	1	2	3	Test
pH of anolyte	2.9 ± 0.1	3.4 ± 0.1	3.9 ± 0.6	–
Degree of irrigation, g of water/100 g of dry substance	190.0 ± 1.9	185.0 ± 1.9	170.0 ± 1.7	180.0 ± 1.8
Hydrothermal resistance after tanning (°C)	66.0 ± 1	65.0 ± 1	57.0 ± 1	64.0 ± 1
Mass content, %, in the skin tissue of unconnected fatty substances	14.2 ± 0.3	14.9 ± 0.3	15.7 ± 0.4	14.3 ± 0.3
Cr_2_O_3_ in skin tissue	1.27 ± 0.03	1.21 ± 0.03	1.14 ± 0.02	1.33 ± 0.03
Breaking stress at the tear of an entire skin (N)	74.0 ± 5.2	79.0 ± 6.6	68.0 ± 5	71.0 ± 5.2
Plasticity (%)	21.0 ± 2.1	19.0 ± 1.9	16.0 ± 1.6	17.0 ± 1.7
Shrinkage of skin tissue (%)	1.2 ± 0.12	1.4 ± 0.14	2.2 ± 0.21	2.8 ± 0.28
Volume yield (cm^3^/100 g)	240.0 ± 12	244.0 ± 13	200.0 ± 10	220.0 ± 11

The volumes of fatty substances and chrome oxide are based on a totally dry substance

The obtained fur skins had somewhat higher plasticity, twofold decrease in the shrinkage of tissue, 10 % increase in volume yield as compared with the existent technology with the exception of Treatment No. 3. Moreover, we observed high chemical activity of anolyte during the manufacture of fur with plastic skin tissue. The achieved degree of watering, namely 185–190 %, can be considered sufficient for forming the skin tissue in subsequent treatments, taking into account that formation of quality fur of bulky sheepskin requires 170–180 g of water per 100 of dry substance even with the use of enzymic preparation (Simonov et al. 1983).

Thus, the use of activated water in preparatory processes of fur manufacture makes it possible to forgo the use of antiseptics as well as of SAMs as well as other soaking enhancers. It also provides for uniting the processes of soaking, degreasing, pickling, tanning and greasing and for cutting the duration of treatments twofold. The use of processing mediums with pH of 3.3–3.5 in acid-salt treatments enhances the effect of electroactivated water on the destruction of links between the macromols of collagen and its other components due to the impact of oxygen-containing free radicals present in water as a result of electrochemical activation.

## Conclusions

The influence of physical/chemical properties of the obtained electroactivated solutions on the processes of soaking, degreasing, pickling, tanning and plasticization in the structuring of tissue of fur skins with different contents of fatty substances was studied.

The influence of processing mediums based on fractions of electroactivated water was established and verified their consistent use for effective processing of fur foregoing antiseptics and surface-active materials.

The use of electrochemically activated water at the stage of restoring the structure of skin tissue collagen and the degree of its hydration accompanied by the release of spheroproteins, carbohydrates and lipids provides for twofold acceleration of the process. The use of the fraction of electroactivated water with high oxidizing power makes the lengthy phase of acid-salt treatment unnecessary.

A low-cost technology of treating furs with different contents of fatty substances was developed, which involves the use of solutions based on electrochemically activated water. The technology foresees substantial economy of water and chemical reagents, a two to threefold accceleration of soaking and tanning processes, and the formation of fur materials with necessary physical/chemical properties. The exclusion of such toxic reagents as formalin and sodium silicofluoride from treatment allows to regard this technology as ecologically safe compared to other technologies of fur manufacture.

## Abbreviations

ECA: electrochemically activated
SAM: surface-active materials
ORP: oxidation–reduction potential
CS: code specification
SAT: statistical acceptance test
T_HS_: hydrothermal stability
